# Vitamin D Deficiency among Patients Visiting a Tertiary Care Hospital: A Descriptive Cross-sectional Study

**DOI:** 10.31729/jnma.5645

**Published:** 2020-11-30

**Authors:** Nimesh Poudel, Subodh Sagar Dhakal, Renu Sukhupayo, Dambar Bahadur Karki

**Affiliations:** 1 Department of Internal Medicine, Kathmandu Medical College and Teaching Hospital, Sinamangal, Kathmandu, Nepal; 2 Department of Cardiology, Kathmandu Medical College and Teaching Hospital, Sinamangal, Kathmandu, Nepal

**Keywords:** *Nepal*, *prevalence*, *vitamin D deficiency*

## Abstract

**Introduction:**

Vitamin D deficiency is a common condition prevalent among both developed and developing countries where it is seen mostly in females. It has been linked to various skeletal and non-skeletal diseases. This study was done to find out the prevalence of Vitamin D deficiency and clinical features of deficient patients attending the outpatient department of a tertiary care hospital.

**Methods:**

This descriptive cross-sectional study was done among the patients attending the outpatient department of a tertiary care hospital in Kathmandu, Nepal. The study was conducted from May 2019 to July 2019. The ethical approval was taken from the Institutional Review Committee (ref no. 310520113). Convenient sampling was done. The collected data was entered in Microsoft Excel and was analyzed in the Statistical Package for the Social Sciences (SPSS) version 26.

**Results:**

Out of 481 participants, the prevalence of vitamin D deficiency was 335 (69.6%) (65.49-73.71 at 95% Confidence Interval). Severe vitamin D deficiency was seen in 78 (16.2%) and insufficient vitamin D in 77 (16%) of the patients. The mean serum vitamin D concentration by gender was 22.38±17.07ng/ml in males and 18.89±15.25ng/ml in females. A total of 263 (54.6%) females and 72 (14.97%) males had vitamin D deficiency. The most common symptoms found in vitamin D deficiency patients were fatigue 187 (55.8%), muscle cramps 131 (39.1%), generalized myalgia 125(37.31%), bone and joint pain 111 (33.13%).

**Conclusions:**

Vitamin D deficiency was prevalent especially in females and elderly people. Fatigability was present in more than half of the vitamin D deficient patients.

## INTRODUCTION

Vitamin D is a fat-soluble prohormone, which is involved in the regulation of the physiological processes.^1^ It is found in two forms; ergocalciferol (Vitamin D2) found in plants and fungi, and cholecalciferol (Vitamin D3) from the sun.^2^ Vitamin D deficiency is defined as “25-hydroxyvitamin D level of less than 20ng per millimeter (50nmol per liter)”.^3,4^

It has been estimated that around one billion people have Vitamin D deficiency or insufficiency.^5^ Vitamin D deficiency shows its high prevalence in the developed countries and the regions of Asia, the Middle East, and India mostly in women.^6^ According to several studies, 40-100% of the US and European population are deficient in Vitamin D.^7,8^ The factors causing Vitamin D deficiency could be due to changes in the lifestyle based on the socio-cultural practice, inadequate sun exposure and the food consumed that are rarely fortified with Vitamin D.^9^ Its deficiency has been linked to different musculoskeletal and non-skeletal complications like congestive heart failure, peripheral vascular disease, hypertension, diabetes mellitus.^10^ So, it is important to know the burden of Vitamin D deficiency for the better patient management.

The present study is done to find out the occurrence of vitamin D deficiency and the frequency of clinical features of vitamin D deficient patients.

## METHODS

This descriptive cross-sectional study was done among patients attending the outpatient department (OPD) and general health checkup of Kathmandu Medical College and Teaching Hospital, Kathmandu, Nepal. The study was conducted from May 2019 to July 2019. The ethical approval was taken from the Institutional Review Committee of the Kathmandu Medical College and Teaching Hospital (ref no. 310520113). Patients visiting the OPDs were included in the study. Patients under the age of 15 years, those with chronic kidney disease and on medication that affects bone metabolism like phenobarbital, anti-tubercular drugs, thiazide, antiretroviral, glucocorticoids, and Vitamin D treatment for Vitamin D deficiency were excluded from the study. Informed written consent was taken from the participants. Convenient sampling was done. The sample size was calculated by using the formula,

$$\begin{array}{ll} \text{n} & \text{= }{\text{Z}}^{\text{2}}\times \text{p}\times \text{(1}-\text{p)}/{\text{e}}^{\text{2}}\\ & \text{= }{\left(1.96\right)}^{\text{2}}\times (0.5)\times (1-0.5)/{(\text{0.07)}}^{\text{2}}\\ & \text{= }196 \end{array}$$

Where,

n = required sample size,
Z = 1.96 at 95% Confidence Interval,
p = population proportion, 50%
e = margin of error, 7%

As patients were enrolled using the convenient sampling, we doubled the size and considering 20% non-respondent rate, the total sample of 481 patients was taken for measurement of vitamin D.

Data collected in semi-structured questionnaire regarding demographics, clinical features like fatigue, body ache, muscle cramps, joint pain, low backache, and medical history of diabetes, hypertension, chronic kidney disease, and Ischemic heart diseases (IHDs). Body mass index was used to define the weight status. Serum Vitamin D3, which is 25 hydroxyvitamin D [25(OH) D], was estimated by a fully-automated chemiluminescence immunoassay.

Vitamin D deficiency was defined as 25(OH) D less than 20ng/ml, Vitamin D insufficiency as 20-29ng/ml, and Vitamin D sufficiency as ≥30ng/ml, and Vitamin D toxicity as more than 100ng/ml. Vitamin D levels less than 10ng/ml were regarded as a severe deficiency.^2,11^

The data collected was entered in Microsoft Excel and was analyzed in the IBM Statistical Package of the Social Sciences (SPSS) version 26. Demographic data and clinical variables were analyzed by descriptive analysis. Results are expressed as mean ± standard deviation for quantitative variables and percentage for qualitative variables like symptoms.

## RESULTS

A total of 481 participants were included in the study. Overall, vitamin D deficiency was found in 335 (69.6%) (65.49-73.71 at 95% Confidence Interval). Insufficiency was seen in 77 (16%) patients. Among the participants, 69 (14.3%) had the sufficient vitamin D levels (Table 1) The total mean age of the participants was 40.5±14.4 years (39.64±14.10 for females and 43.59±15.13 for males). The majority were females with a female: male ratio of 3.3:1. The study included 111 (23.1%) males and 370 (76.1 %) females.

**Table 1:** Vitamin D status in patients.

Vitamin D levels (ng/ml)	Frequency n (%)
Deficiency (< 20)	335 (69.6)
Insufficiency (≥20 -<30)	77 (16)
Sufficiency (≥30)	69 (14.3)
Total	481 (100)

The total mean serum vitamin D was 19.69±13.68ng/ ml. The mean serum vitamin D concentration by gender was 22.38±17.07ng/ml in males and 18.89±15.25ng/ ml in females.

In the study, out of 481 participants, 78 (16.2%) of the participants had severe vitamin D deficiency. Among severely vitamin D deficient patients, 59 (75.6%) were symptomatic. The most common symptoms among them were fatigability 44 (56.4%), generalized myalgia 41 (52.6%), bone and joint pain 35 (44.9%), and muscle cramps 37 (47.4%). Among 257 mild vitamin D deficient patients, 169 (65.8%) participants had symptoms. The symptoms among them were fatigability 143 (55.6%), muscle cramps 94 (36.6%), generalized myalgia 84 (32.7%), bone and joint pain in 76 (29.6%). Overall, 227(68.05%) of vitamin D deficient patients were symptomatic. Vitamin D deficient patient includes mild and severe vitamin D deficiency. The symptoms among vitamin D deficient were fatigability 187 (55.8%), muscle cramps 131 (39.1%), generalized myalgia 125 (37.31%), bone and joint pain 111 (33.13%). The symptoms were predominantly seen in patients with severe deficiency. Some patients with severe deficiency also have a history of hair fall (Table 2).

**Table 2:** Symptoms among patients with severe vitamin D deficiency and deficiency.

Symptoms	Severe Deficiency (n = 78) n (%)	Deficiency (n = 335) n (%)
Fatigability	44 (56.4)	187 (55.8)
Generalized Myalgia	41 (52.6)	125(37.31)
Muscle cramps	35 (44.9)	131 (39.1)
Bone and joint pain	37 (47.4)	111(33.13)

A total of 263 (54.6%) females and 72 (14.97%) males had vitamin D deficiency (Table 3).

**Table 3:** Status of Vitamin D among males and females (n = 481).

		Gender	
		Male n (%)	Female n (%)
Vitamin D levels in ng/ml	<20	72 (15.0)	263 (54.7)
	≥20 - <30	15 (3.1)	62 (12.9)
	≥30	24 (5.0)	45 (9.4)

Seventy-six (22.7%) of patients with vitamin D deficiency were between the 66-75 aged group followed by 36-45 aged groups with 68 (20.3%) patients, 26-35 aged groups with 63 (18.8%) patients, and 46-55 aged groups with 57 (17%) patients. The age group with the least number of patients with vitamin D deficiency was between 15-25 aged groups with 9 (1.87%).

## DISCUSSION

The results of this cross-sectional study done in a tertiary care hospital of Kathmandu, Nepal showed the prevalence of Vitamin D deficiency as 69.6%, of insufficiency as 16%, and sufficient Vitamin D in 14.3%. The prevalence was higher among older ages and females. A severe deficiency was seen in 16.2% of the studied population. The rates of Vitamin D deficiency found in this study are markedly higher than in many western countries like in Germany, Austria, and the Netherlands, in North Europe (Denmark, Finland, Ireland, and Poland), Canada, and the United Kingdom which have shown the prevalence of vitamin D deficiency from 10-55.5%.^11,12^

Mariam Omar et al. reported a deficiency of 76.1% and insufficiency of 15.2% among the population of Benghazi, a sunny second-largest city in the east of Libya.^2^ Our results share similar vitamin D deficiency status with some parts of Africa, Asia, and the Middle East. The prevalence of vitamin D deficiency in Egypt was 77%, insufficiency was 15%, and 9% of the population has sufficient Vitamin D levels. In Qatar, 83-91% of the population is deficient in Vitamin D.^2,13^

Vitamin D deficiency is considered to be a public health problem worldwide. Female gender is one of the most important predictors of vitamin D deficiency.^2^ In this study, 23.1% of the participants were males and 76.1% were females. Out of the studied population, 54.7% of the females and 15% of the males had vitamin D deficiency. This finding of increased prevalence seen in females is comparable to other studies and can be due to a sedentary lifestyle and aggressive sun protection. The greater participation of females in the studies may be due to the greater willingness of females to use health services.^2^ Babita Ghai et al. reported 73% of the female to be vitamin D deficient.^14^ In contrast, Manoharan et al. studied the vitamin D status among people of Tamil Nadu and reported that 46% of the males and 37% of the females had a vitamin D deficiency.^15^

Vitamin D deficiency was seen in 22.7% of patients between the 66-75 aged group and 20.3% in the 36-45 age group and 17% in 46-55 aged group patients. Various studies report a similar observation by demonstrating lower vitamin D levels with increasing ages and higher vitamin D deficiency states in the older age group, mandating early investigating and thus helping them to prevent falls and fractures.^2,16^

In the study, it was found that among the 335 vitamin D deficient patients, 228 (68.05%) of the participants had symptoms. The most common symptoms that led the participant to seek health services were fatigue, generalized myalgia, and bone pain. Fatigability was seen in 55.8%, muscle cramps in 39.1%, generalized myalgia in 39.1%, bone and joint pain in 33.13% of the vitamin D deficient patients. The symptoms were predominantly seen in patients with severe deficiency. Lubna M et al, in their study, showed that the most common reason for requesting vitamin D level included generalized myalgia and bone pain in 51% of the patients.^17^ Satyajeet Roy et al. showed the prevalence of low vitamin in inpatients as 77.2% with fatigue and fatigability was normalized after correction of vitamin D level.^18^

Our study has demonstrated a high prevalence of vitamin D deficiency among patients visiting in our center. A larger multicentric or community based study with a diverse sample population should be conducted in the future to find out a more accurate prevalence. Similarly, other studies that further look into the association between gender and age and other comorbidities with vitamin D levels in Nepalese are warranted.

## CONCLUSIONS

Vitamin D deficiency was prevalent especially in females and elderly people. Fatigability was present in more than half of the vitamin D deficient patients. Muscle cramps and generalized myalgia were present in more than one third and bone and joint pain in almost one third of the vitamin D deficient patients.
